# The impact of hypertension on clinical manifestations of Omicron variant BA.1 infection in adult patients

**DOI:** 10.1128/spectrum.04168-23

**Published:** 2024-04-26

**Authors:** Jingyu Wang, Henan Dong, Jie Zhao, Chunlei Zhou, Meng Wang, Yuechuan Cui, Guangfeng Gao, Xiaodong Ji, Hong Mu, Lin Peng

**Affiliations:** 1 Department of Laboratory Medicine, Tianjin First Central Hospital, Tianjin, China; 2 The First Central Clinical School, Tianjin Medical University, Tianjin, China; 3 Clinical Medical School, Jiamusi University, Jiamusi, Heilongjiang, China; 4 Department of Radiology, Tianjin First Central Hospital, Tianjin, China; Oklahoma State University College of Veterinary Medicine, Stillwater, Oklahoma, USA

**Keywords:** COVID-19, SARS-CoV-2, hypertension, Omicron BA.1, clinical manifestations

## Abstract

COVID-19 caused by Omicron BA.1 has resulted in a global humanitarian crisis. In this COVID-19 pandemic era, hypertension has been receiving increased attention. Omicron BA.1 infection combined with hypertension created a serious public health problem and complicated the treatment and prognosis of COVID-19. The aim of our study was to assess the implications of hypertension for the clinical manifestations of adult patients (APs) infected with Omicron BA.1. This single-center retrospective cohort study enrolled consecutive COVID-19 APs, who were admitted to Tianjin First Central Hospital from 01 August 2022 to 30 November 2022. All included APs were divided into two groups: hypertension and non-hypertension group. The APs’ baseline demographic, laboratory, clinical, and radiological characteristics were collected and analyzed. Of 512 APs admitted with PCR proven COVID-19, 161 (31.45%) APs had comorbid hypertension. Hypertension APs have older age, higher body mass index, lower Ct-values of the viral target genes at admission, and longer hospital stay than non-hypertension APs. Furthermore, hypertension aggravates the clinical classification, impairs liver, kidney, and myocardium function, and abnormalizes the coagulation system in Omicron BA.1- infected APs. Moreover, hypertension elevates inflammation levels and lung lesion involvement while weakened virus-specific IgM level in APs with Omicron BA.1 infection. Hypertension APs tend to have worse clinical conditions at baseline than those non-hypertension APs. This study indicates that hypertension is a contributor to the poor clinical manifestations of Omicron BA.1-infected APs and supports that steps to control blood pressure should be a vital consideration for reducing the burden of Omicron BA.1 infection in hypertension individuals.

**IMPORTANCE:**

This study provided inclusive insight regarding the relationship between hypertension and Omicron BA.1 infection and supported that hypertension was an adverse factor for COVID-19 APs. In conclusion, this study showed that hypertension was considered to be associated with severe conditions, and a contributor to poor clinical manifestations. Proper medical management of hypertension patients is an imperative step in mitigating the severity of Omicron BA.1 variant infection.

## INTRODUCTION

COVID-19 was a pandemic disease first detected in Wuhan, China, and caused by SARS-CoV-2, a novel single-stranded RNA virus. Globally, as of 31 December 2023, there have been 773,819,856 reported cases of COVID-19, including 7,010,568 deaths (https://covid19.who.int/). Despite the phylogenetic and clinical trial similarities between SARS-CoV-2 and SARS-CoV, this novel coronavirus exhibits a higher transmission rate and a lower mortality rate (1). SARS-CoV-2 binds the host cell through the receptor-binding domain (RBD) with its S1 subunit to the angiotensin-converting enzyme 2 (ACE2) receptor expressed on the host cell membranes (2, 3). ACE2 is extensively expressed in various organ systems of the human body, such as the respiratory system and the cardiovascular system (4). The World Health Organization (WHO) categorizes the circulating COVID-19 variants into Alpha (B.1.1.7), Beta (B.1.351), Gamma (P.1), Delta (B.1.617.2), and Omicron (B.1.1.529) (5). Among these variants, Omicron variant BA.1, a descendant lineage of B.1.1.529, was the dominant strain in the global COVID-19 pandemic, causing global panic and retarding social and economic development.

SARS-CoV-2 infections caused mild disease in most individuals. About 10%–20% of COVID-19 patients progress to a severe course of disease with pneumonia and respiratory failure (6). Since the onset of the pandemic, multiple comorbidities, including obesity, diabetes, and coronary heart disease, have increased the risk of dying in patients with COVID-19 (7, 8). A previous study has suggested that hypertension may be one of the most common comorbidities in COVID-19 patients (9). Another clinical study indicated a prevalence of hypertension of 23.4% in severe COVID-19 patients (10). Although hypertension may not be a common feature as a risk factor for severity, the need to assess its contribution in the severity and mortality of the infection has been highlighted in a recent analysis of 17 million patients (11).

The ACE2, as the cell surface receptor for SARS-CoV-2 infection, plays a pivotal role in normal physiological activities of the human body and may protect against lung injury in infection (12). Several views suggested that underlying chronic disease, such as hypertension, in comorbid conditions might lead to the upregulation of certain ACE2-, CD26-, and CD147-related molecules, potentially increasing the susceptibility of patients to SARS-CoV-2 infection (13). However, it remains ill-defined whether hypertension is an exact risk factor for Omicron BA.1-infected APs. Furthermore, there is a lack of clinical characteristics analysis of hypertension APs infected with Omicron BA.1 in China. Therefore, evidences of the impact of hypertension on APs infected with Omicron BA.1 are urgently needed, and the clinical manifestations of hypertension APs infected with Omicron BA.1 need to be revealed.

To further investigate whether hypertension is a detrimental factor affecting the progress and prognosis of Omicron BA.1 infection and avoid the confounding effects of other underlying diseases, we excluded APs with underlying diseases other than hypertension and finally enrolled 512 consecutive APs admitted to Tianjin First Central Hospital, which is a hospital dedicated solely to the treatment of COVID-19, in Tianjin, China. Their clinical manifestations and laboratory parameters were collected and analyzed. In brief, we rigorously focused on and comprehensively analyzed the effects of hypertension on clinical characteristics of Omicron BA.1-infected APs. This study not only strengthened the comprehension of the effects of hypertension on the clinical characteristics of COVID-19 APs but also provided a valuable reference for the prevention and treatment of Omicron BA.1 infection. Additionally, this research might be beneficial to governments in developing prevention and treatment policies and responding to the Omicron variant BA.1 pandemic.

## MATERIALS AND METHODS

### Study design

The present study was a retrospective observational study. A total of 3,088 consecutive COVID-19 patients diagnosed with Omicron BA.1 infection, who were admitted to Tianjin First Central Hospital (Tianjin, China) from 01 August 2022 to 30 November 2022, were enrolled. This hospital was one of the hospitals designated by the Chinese government to hospitalize COVID-19 patients during the pandemic. All included patients were diagnosed with COVID-19 according to the Diagnosis and Treatment of Novel Coronavirus Pneumonia guidelines (9th edition) issued by the National Health Commission of China (https://www.gov.cn/zhengce/zhengceku/2022-03/15/content_5679257.htm) and confirmed by reverse transcription polymerase chain reaction (RT-PCR). RT-PCR following the WHO protocol to detect two target genes, the open reading frame of 1ab (ORF1ab) and the nucleocapsid protein (N). A cycle threshold value (Ct-value) less than 37 with an S-shape amplification curve was defined as positive. Daily follow-up in person and SARS-CoV-2 RNA detection were performed simultaneously. The clinical classifications for COVID-19 patients were based on the New Coronavirus Pneumonia Prevention and Control Program (9th edition), published by the National Health Commission of China (http://www.gov.cn/zhengce/zhengceku/2022-03/15/content_5679257.htm). The severity of SARS-CoV-2 infection was categorized as (1) mild, if clinical symptoms were mild, and there was no evidence of pneumonia on imaging; (2) moderate, if pneumonia was accompanied by fever and respiratory tract symptoms, and there were imaging features of pneumonia; (3) severe, if the respiratory rate was ≥30/min, oxygen saturation was ≤93% when breathing ambient air, or PaO_2_/FiO_2_ ≤ 300 mm Hg (1 mm Hg = 0.133 kPa); or (4) critical, the clinical symptoms were progressively aggravated, and the lung imaging showed that the lesions significantly progressed more than 50% within 24–48 h.

### Inclusion and exclusion criteria

**Fig 1:**
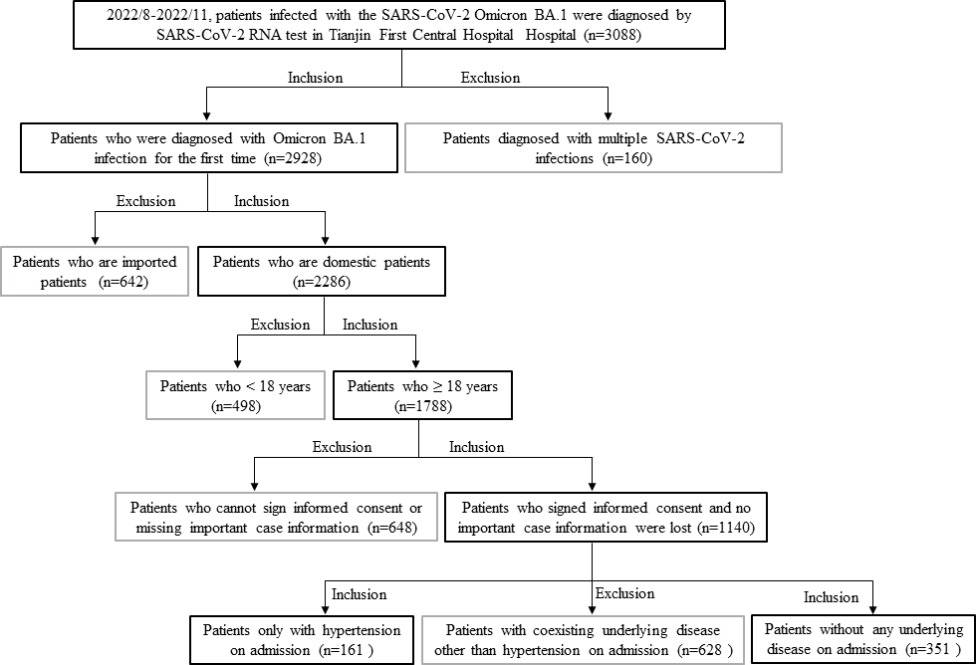
The flow chart of the critical of cases inclusion or exclusion.

The workflow for the inclusion, exclusion, and grouping of patients is presented in Fig. 1. Inclusion criteria: (1) meeting the SARS‐CoV‐2 positive diagnostic criteria on admission; (2) local COVID-19 patients admitted to Tianjin, China (non-imported patients); (3) patients are older than 18 years; (4) patients diagnosed with no underlying medical disease or only hypertension on admission; (5) patients diagnosed with Omicron BA.1 infection for the first time. Exclusion criteria: (1) patients diagnosed with negative SARS‐CoV‐2 pathogen test on admission; (2) imported patients; (3) patients < 18 years; (4) patients with coexisting underlying disease other than hypertension on admission; (5) patients who are unable to sign informed consent forms or omit important case information. The diagnosis of hypertension was based on the documented medical history of hypertension, with a systolic blood pressure of ≥140 mmHg or a diastolic blood pressure of ≥90 mmHg. Eventually, this study conducted an in-depth analysis of clinical data of 512 APs infected with Omicron BA.1.

### Chest CT parameters and evaluation

**Fig 2:**
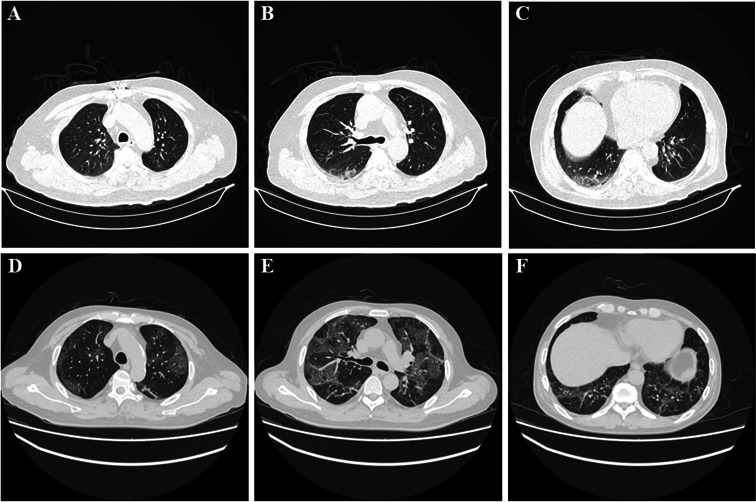
Chest CT image of APs with Omicron BA.1 infection on admission. (A–C) Chest CT scan of non-hypertensive patient, showing small patchy ground glass opacity in the left upper lobe and the right lower lobe; (D–F) chest CT scan of hypertensive patient, showing multiple patchy ground glass opacity and crazy-paving pattern in both lungs.

Chest CT scans were performed using 64-MDCT (the MX-64 Slice scanner) (Somatom Definition AS). The CT images were acquired using the following parameters: tube voltage, 120 kVp; tube current, 200–280 mA; reconstruction matrix size, 512 × 512; slice thickness, 1.5 mm. In order to comprehensively quantify and evaluate the severity of the lung lesion on the chest CT scans, the scoring criteria developed by Huang et al. (14) were applied, which takes into account the extent of involvement and CT manifestations of lung lesions, in order to effectively achieve the semi-quantification of lesions. The scoring of the chest CT scans was performed by two senior radiologists (G.G. and X.J.). Disagreements were resolved by consensus. Figure 2 presents two examples of chest CT images of APs with Omicron BA.1 infection.

### Data acquisition

The medical records were examined by the HIS system of Tianjin First Central Hospital to obtain the general data, medical history, clinical manifestations, laboratory results, clinical symptoms, and comorbidity on admission of APs. Chest computed tomography (CT) images were extracted from picture archives and communication systems. Clinical and laboratory data were collated for each enrolled patient prior to discharge.

### Ethics

This study was approved by the Medical Ethics Committee of Tianjin First Central Hospital (No. 2022N052KY) and was conducted in accordance with the Declaration of Helsinki. All data used in the study were anonymized, and all participants signed written informed consent to participate in and publish this study. The data set analyzed in the present study is available from the corresponding author on reasonable request.

### Statistical analysis

SPSS 25.0 (IBM, Inc, Chicago, IL, USA) software was used for statistical analysis without filling in the missing data. Continuous variables are expressed as mean ± standard deviation or median of the interquartile range (if appropriate), and categorical variables are expressed as frequency (percentage). When the data are normally distributed, use the unpaired *t*-test to compare significant differences in continuous variables; Otherwise, use the Mann-Whitney *U*-test. The counting data were compared using the *χ*2 test or the Fisher exact probability method. The difference in *P* < 0.05 was statistically significant.

## RESULTS

### Demographics and basic clinical characteristics of participants

The study flowchart showing the strategy of case inclusion or exclusion is illustrated in Fig. 1. A total of 3,088 patients with Omicron BA.1 infection were diagnosed in the Tianjin First Central Hospital (Tianjin, China) between August and November 2022. Patients who were diagnosed with multiple SARS-CoV-2 infections (*n* = 160) or imported patients (*n* = 642) or aged <18 years (*n* = 498) or with coexisting underlying diseases other than hypertension on admission (*n* = 628) or unable to sign informed consent or missing important case information (*n* = 648) are excluded. Five hundred twelve APs infected with Omicron BA.1 are enrolled in this study finally, including 351 (68.55%) non-hypertension APs and 161 (31.45%) hypertension APs. The demographics and baseline clinical characteristics are presented in Table 1. The median age of 512 APs was 40 (29–53) years old. The age of 351 non-hypertension APs was 35 (24–44) years old, including 185 (52.71%) males and 166 (47.29%) females, and the age of 161 hypertension APs was 53 (41–60) years, including 104 (64.60%) males and 57 (35.40%) females. The age of hypertension APs was significantly exceeded that of non-hypertension APs (*P* < 0.001). The body mass index (BMI) of 512 APs was 24.71 (21.72–27.68), and hypertension APs [26.53 (24.30–29.27)] had higher BMI levels than non-hypertension APs [23.88 (21.26–26.70), *P* < 0.001]. Additionally, 332 (94.59%) non-hypertension APs and 158 (98.14%) hypertension APs received the SARS-CoV-2 vaccine. There was no discernible differences in vaccination rates between the two groups. Further comparatively analyzed the clinical symptoms between the two groups. As shown in Table 1, the most common clinical symptom of all patients was fever, which was observed in 32.23% APs. It is noteworthy that the percentages of patients who experienced cough, expectoration, and fatigue are significantly higher among hypertension APs compared to non-hypertension APs. Additionally, compared with non-hypertension APs, hypertension APs had notable lower Ct-values of the SARS-CoV-2 viral target genes (N gene and ORF1ab gene) detected by RT-PCT at admission and obvious longer hospital stay.

**TABLE 1:** Baseline characteristics of APs infected with Omicron BA.1 on admission^*a*^

	APs (*n* = 512)	Non-hypertension APs (*n* = 351)	Hypertension APs (*n* = 161)	*P*-value
Age, years	40 (29–53)	35 (24–44)	53 (41–60)	<0.001
Gender				NA^*b*^
Male	289 (56.45)	185 (52.71)	104 (64.60)	
Female	223 (43.55)	166 (47.29)	57 (35.40)	
BMI	24.71 (21.72–27.68)	23.88 (21.26–26.70)	26.53 (24.30–29.27)	<0.001
Vaccination				0.066
Unvaccinated	22 (4.30)	19 (5.41)	3 (1.86)	
Vaccinated	490 (95.70)	332 (94.59)	158 (98.14)	
Clinical symptoms				
Fever	165 (32.23)	114 (32.48)	51 (31.68)	0.857
Stuffy nose	24 (4.69)	17 (4.84)	7 (4.35)	0.805
Runny nose	23 (4.49)	18 (5.13)	5 (3.11)	0.305
Cough	116 (22.66)	70 (19.94)	46 (28.57)	0.030
Expectoration	58 (11.33)	33 (9.40)	25 (15.53)	0.042
Fatigue	52 (10.16)	28 (7.98)	24 (14.91)	0.016
Sore throat	91 (17.77)	65 (18.52)	26 (16.15)	0.515
Allotriosmia	3 (0.59)	1 (0.28)	2 (1.24)	0.234
Allotriogeustia	3 (0.59)	2 (0.57)	1 (0.62)	1.000
Headache	49 (9.57)	39 (11.11)	10 (6.21)	0.080
Nausea and vomiting	19 (3.71)	15 (4.27)	4 (2.48)	0.320
Diarrhea	5 (0.98)	5 (1.42)	0 (0.00)	0.299
Virus target genes Ct-value				
N gene	22.03 (18.85–27.09)	22.43 (19.16–27.73)	20.95 (18.22–24.83)	0.012
ORF1ab gene	25.66 (22.81–30.47)	26.15 (23.14–31.49)	24.51 (21.63–28.82)	0.001
Hospital length of stay, days	13.64 (10.53–17.59)	13.20 (9.45–16.45)	15.45 (11.86–18.75)	<0.001

^*a*^ Data are presented as median (IQR) or *n *(%). *n* is the number of patients with available data. Percentages may not total 100 because of rounding. *P* < 0.05 were statistically significant.

^*b*^ NA, not applicable; BMI, body mass index.

### Clinical classification of APs at diagnosis

The clinical classifications for COVID-19 were based on the New Coronavirus Pneumonia Prevention and Control Program (9th edition), published by the National Health Commission of China (http://www.gov.cn/zhengce/zhengceku/2022-03/15/content_5679257.htm). As indicated in Table 2, hypertension group had a significantly lower proportion of asymptomatic conditions while a visible higher proportion of mild and moderate conditions compared to non-hypertension group (*P* < 0.05). Furthermore, a logistic regression analysis was conducted to explore the risk factors of COVID-19 disease severity (Table 3). The analysis revealed that hypertension and the higher levels of neutrophils counts, CRP, CK-MB, NT-proBNP, CREA, and GGT were important adverse factors to aggravate the severity of Omicron BA.1 infection, while the higher lymphocytes level was an independent protective factor for relieving the severity of the Omicron BA.1 infection. These findings suggest that hypertension is a detrimental factor that aggravates the severity of Omicron BA.1 infection.

**TABLE 2:** Clinical classifications of APs infected with Omicron BA.1 at admission^*a*^

	APs (*n* = 512)	Non-hypertension APs (*n* = 351)	Hypertension APs (*n* = 161)	*P*-value
Asymptomatic	331 (64.65)	256 (72.93)	75 (46.58)	<0.001
Mild	115 (22.46)	69 (19.66)	46 (28.57)	0.025
Moderate	65 (12.70)	26 (7.41)	39 (24.22)	<0.001
Severe	1 (0.20)	0 (0.00)	1 (0.62)	0.314

^*a*^ Data are presented as *n *(%). *n* is the number of patients with available data. Percentages may not total 100 because of rounding. *P* < 0.05 were statistically significant.

**TABLE 3:** Binary logistic regression analysis^*a*^

	Univariate regression analysis		
Variables	OR	95% CI	*P*-value
Age	1.008	0.969, 1.048	0.694
Gender	0.939	0.220, 4.006	0.932
Hypertension	2.883	1.019, 8.159	0.046
Neutrophils	2.183	1.339, 3.558	0.002
Lymphocytes	0.034	0.003, 0.344	0.004
NLR	0.956	0.751, 1.217	0.714
IL-6	1.055	0.987, 1.128	0.114
CRP	1.114	1.052, 1.181	<0.001
ALT	1.005	0.968, 1.043	0.808
AST	1.048	0.989, 1.111	0.110
GGT	1.037	1.012, 1.063	0.004
CREA	1.047	1.006, 1.090	0.025
UREA	1.126	0.688, 1.843	0.637
CK-MB	1.795	1.134, 2.841	0.012
cTn	1.059	0.985, 1.138	0.120
NT-proBNP	1.005	1.001, 1.008	0.013

^*a*^ OR, odds ratio; CI, confidence interval; NLR, neutrophil to lymphocyte ratio; CRP, C reaction protein; ALT, alanine aminotransferase; AST, aspartate aminotransferase; GGT, gamma-glutamyltransferase; CREA, creatinine; UREA, urea; CK-MB, creatine kinase-MB; cTn, cardiac troponin; NT-proBNP, N-terminal pro-B-type natriuretic peptide.

### Characteristics of liver, kidney, and myocardium function-associated indicators

As depicted in Table 4, the laboratory data of liver, kidney, and myocardium function-associated indicators were collected and subjected to analysis. Compared with non-hypertension group, hypertension APs had visibly higher levels of alanine aminotransferase (ALT), aspartate aminotransferase (AST), gamma-glutamyltransferase (GGT), lactic dehydrogenase (LDH), total bilirubin (TBIL), indirect bilirubin (IBIL), and direct bilirubin (DBIL) (*P* < 0.05). Furthermore, the comparative analysis of the renal function-associated indicators was conducted between hypertension and non-hypertension APs. Hypertension APs had significantly higher urea (UREA), creatinine (CREA), Uric Acid (UA), and UREA/CREA levels than non-hypertension APs. As for the myocardium function indicators, the creatine kinase-MB (CK-MB), myoglobin (Mb), cardiac troponin (cTn), and N-terminal pro-B-type natriuretic peptide (NT-proBNP) levels were markedly higher in hypertension APs than those in non-hypertension APs. These results indicate that hypertension APs tend to show organ injury more than non-hypertension APs, which may lead to a poor prognosis of COVID-19 patients.

**TABLE 4:** Liver, renal, and myocardial function parameters of APs**^*a*^**

	APs (*n* = 512)	Non-hypertension APs (*n* = 351)	Hypertension APs (*n* = 161)	*P*-value
Liver function				
ALT (U/L)^*b*^	20.55 (14.47–30.98)	18.41 (13.79–29.39)	25.89 (17.59–35.59)	<0.001
AST (U/L)	23.98 (19.66–29.86)	22.41 (19.05–28.71)	26.19 (22.12–33.47)	<0.001
ALP (U/L)	77.28 (62.99–93.41)	75.07 (61.55–93.06)	80.89 (66.22–97.75)	0.067
GGT (U/L)	20.95 (15.30–36.85)	19.22 (14.52–32.23)	29.43 (19.00–44.77)	<0.001
LDH (U/L)	182.94 (163.10–207.54)	178.14 (158.52–202.40)	196.10 (174.89–214.18)	<0.001
TBIL (μmol/L)	10.35 (7.61–14.09)	9.67 (7.25–13.33)	11.60 (8.43–17.07)	<0.001
IBIL (μmol/L)	6.63 (4.47–10.00)	6.16 (4.28–9.16)	7.53 (4.69–11.15)	0.018
DBIL (μmol/L)	3.77 (2.66–4.91)	3.52 (2.56–4.40)	4.58 (3.33–5.78)	<0.001
Renal function				
UREA (mmol/L)	4.25 (3.60–5.13)	3.97 (3.45–4.75)	4.71 (3.95–5.81)	<0.001
CREA (μmol/L)	66.63 (53.56–79.71)	62.88 (51.29–77.94)	72.56 (62.55–82.83)	<0.001
UA (μmol/L)	336.12 (272.62–404.10)	324.51 (259.75–394.66)	355.41 (302.61–418.21)	0.001
UREA/CREA	0.06 (0.05–0.08)	0.06 (0.05–0.08)	0.07 (0.06–0.08)	0.037
Myocardium function				
CK-MB (ng/mL)	1.05 (0.67–1.61)	0.88 (0.61–1.37)	1.32 (0.86–2.05)	<0.001
Mb (ng/mL)	32.04 (25.54–43.57)	28.87 (24.58–37.38)	36.49 (28.66–53.68)	<0.001
cTn (ng/L)	8.04 (6.33–11.40)	7.50 (5.94–10.09)	10.43 (7.33–14.08)	<0.001
NT-proBNP (pg/mL)	60.83 (24.85–122.43)	51.44 (21.19–99.29)	93.24 (32.73–195.20)	<0.001

^*a*^ Data are presented as median (IQR) or *n *(%). *n* is the number of patients with available data. Percentages may not total 100 because of rounding. *P* < 0.05 were statistically significant.

^*b*^ ALT, alanine aminotransferase; AST, aspartate aminotransferase; ALP, alkaline phosphatase; GGT, gamma-glutamyltransferase; LDH, lactic dehydrogenase; TBIL, total bilirubin; IBIL, indirect bilirubin; DBIL, direct bilirubin; UREA, urea; CREA, creatinine; UA, uric acid; CK-MB, creatine kinase-MB; Mb, myoglobin; cTn, cardiac troponin; NT-proBNP N-terminal pro-B-type natriuretic peptide.

### Characteristics of blood coagulation function-associated biomarkers

The impact of COVID-19 on the cardiovascular system has been a topic of growing interest and concern among medical professionals worldwide. Recent researches have uncovered the potential link between the viral infection and various cardiac events such as thrombosis. It is widely accepted that SARS-CoV-2 causes dysregulation of coagulation processes (15). Furthermore, most therapies for COVID-19 patients target either the immune system or coagulation processes. As the inflammation and coagulation systems are highly connected, a growing number of researches have shown the observed dysregulation of the coagulation system in COVID-19 patients (16–18). Therefore, we furtherly explored the effect of hypertension on the coagulation system of APs infected with Omicron BA.1. Table 5 showed the differences in blood coagulation function-associated parameters of APs between the two groups. Compared with non-hypertension APs, hypertension APs had visible lower levels of activated partial thromboplastin time (APTT) and prothrombin time (PT). Similarly, we also observed that hypertension APs had a significantly lower international normalized ratio (INR) than non-hypertension APs. Nevertheless, the prothrombin activity (PTA) level was higher in hypertension APs than in non-hypertension APs. There was no significant differences in thrombin time (TT), fibrinogen (Fg), and D-Dimer levels between the two groups (*P* > 0.05). These results suggest that hypertension has significant adverse effects on the coagulation system of Omicron BA.1-infected APs, which might result in patients being in a procoagulant disease state.

**TABLE 5:** Laboratory parameters of blood coagulation function in different APs group^*a*^

	APs (*n* = 512)	Non-hypertension APs (*n* = 351)	Hypertension APs (*n* = 161)	*P*-value
APTT (s)^*b*^	32.40 (29.70–35.10)	33.10 (30.45–35.98)	30.65 (28.60–34.00)	<0.001
PT (s)	12.70 (12.20–13.60)	12.90 (12.30–13.90)	12.40 (12.00–13.00)	<0.001
TT (s)	18.20 (17.70–18.70)	18.10 (17.60–18.70)	18.20 (17.80–18.70)	0.565
Fg (g/L)	2.76 (2.42–3.18)	2.72 (2.41–3.17)	2.77 (2.45–3.18)	0.297
INR	1.02 (0.98–1.10)	1.04 (0.99–1.13)	1.00 (0.96–1.05)	<0.001
PTA (%)	95.50 (82.20–104.80)	92.20 (78.60–102.80)	100.90 (90.60–109.10)	<0.001
D-Dimer (mg/L)	0.28 (0.17–0.49)	0.27 (0.16–0.49)	0.30 (0.17–0.46)	0.652

^*a*^ Data are presented as median (IQR) or *n *(%). *n* is the number of patients with available data. Percentages may not total 100 because of rounding. *P* < 0.05 were statistically significant.

^*b*^ APTT, activated partial thromboplastin time; PT, prothrombin time; TT, thrombin time; Fg, fibrinogen; INR, intertional normalized ratio; PTA, prothrombin activity.

### Differences in inflammation and immune-associated biomarkers between non-hypertension and hypertension APs

The immune system plays a pivotal role in antiviral infection and rehabilitation. To further investigate the effects of hypertension on the immune characteristics of APs infected with Omicron BA.1, the inflammatory and immune-related biomarkers were reviewed and analyzed. As shown in Table 6, the levels of higher white blood cells (WBCs), neutrophils, and basophils were significantly elevated compared to those in non-hypertension APs, while no obvious difference was observed in other immune cells between the two groups. Additionally, the level of IL-6, a key inflammation biomarker, was significantly higher in hypertension APs than that in non-hypertension APs. We observed a similar phenomenon in C-reactive protein (CRP), another important inflammation biomarker, between the two groups although there was no significant difference (*P* = 0.090). Additionally, virus-specific IgM level in hypertension APs was notably lower than that in non-hypertension APs, while virus-specific IgG level showed no significant differences between the two groups. These findings suggested that hypertension not only aggravated inflammation levels but also weakened IgM level in Omicron BA.1-infected APs, which would be a risk factor for poor prognosis.

**TABLE 6:** The inflammation and immune-related parameters of APs infected with Omicron BA.1^*a*^

	APs (*n* = 512)	Non-hypertension APs (*n* = 351)	Hypertension APs (*n* = 161)	*P*-value
WBCs (10^9^/µL)^*b*^	5.84 (4.72–7.14)	5.71 (4.62–7.06)	6.09 (4.98–7.47)	0.027
Neutrophils (10^9^/µL)	3.96 (2.98–5.22)	3.81 (2.94–5.07)	4.23 (3.29–5.36)	0.027
Lymphocytes (10^9^/µL)	1.06 (0.69–1.52)	1.05 (0.68–1.52)	1.06 (0.73–1.49)	0.662
NLR (%)	3.81 (2.13–6.25)	3.78 (2.04–6.17)	4.15 (2.29–6.51)	0.356
Monocytes (10^9^/µL)	0.53 (0.41–0.66)	0.51 (0.41–0.65)	0.54 (0.43–0.69)	0.320
Eosinophils (10^9^/µL)	0.03 (0.01–0.08)	0.03 (0.01–0.08)	0.03 (0.01–0.07)	0.694
Basophils (10^9^/µL)	0.02 (0.01–0.02)	0.01 (0.01–0.02)	0.02 (0.01–0.03)	0.014
IL-6 (pg/mL)	10.04 (7.09–15.15)	9.42 (6.68–14.42)	11.30 (8.15–16.23)	0.008
CRP (mg/L)	4.79 (1.69–10.47)	4.56 (1.47–9.71)	5.21 (2.32–11.72)	0.090
IgG (S/CO)	4.98 (1.22–14.21)	4.98 (1.20–14.38)	4.93 (1.27–13.94)	0.954
IgM (S/CO)	0.11 (0.07–0.24)	0.12 (0.07–0.26)	0.10 (0.07–0.18)	0.027

^*a*^ Data are presented as median (IQR) or *n *(%). *n* is the number of patients with available data. Percentages may not total 100 because of rounding. *P* < 0.05 were statistically significant.

^*b*^ WBCs, white blood cell counts; NLR, neutrophil to lymphocyte ratio; CRP, C reaction protein.

### Chest CT features of hypertension APs

As a respiratory infection virus, the lung is the first organ affected by the SARS-CoV-2. The differences of chest CT features, including the extent and distribution of lesion involvement, predominant radiologic pattern, and scores for the lung, between the two groups are presented in Table 7. The proportion of hypertension APs with bilateral lung involvement lesions was significantly higher than the non-hypertension group. Additionally, the proportion of individuals with GGO, patchy shadow, and cord-like density shadow in hypertension APs was obvious higher than that in non-hypertension APs. Observably, compared with non-hypertension APs, hypertension APs had higher chest CT scores of left lung, right lung, and whole lung. On the whole, compared with non-hypertension group, the lung lesion involvement in hypertension APs was more significantly worse.

**TABLE 7:** Chest CT findings and score**s^*a*^**

	APs (*n* = 404)	Non-hypertension APs (*n* = 295)	Hypertension APs (*n* = 109)	*P*-value
Pneumonia				
Normal	97 (24.01)	86 (29.15)	11 (10.09)	<0.001
Unilateral (left)	41 (10.15)	35 (11.86)	6 (5.50)	0.060
Unilateral (right)	76 (18.81)	62 (21.02)	14 (12.84)	0.062
Bilateral (both)	190 (47.03)	112 (37.97)	78 (71.56)	<0.001
Predominant CT pattern				
Ground-glass opacity (GGO)	124 (30.69)	78 (26.44)	46 (42.20)	0.002
Patchy shadow	38 (9.41)	21 (7.12)	17 (15.60)	0.010
Cord-like density shadow	147 (36.39)	86 (29.15)	61 (55.96)	<0.001
Consolidation	178 (44.06)	122 (41.36)	56 (51.38)	0.072
Linear density shadows	14 (3.47)	9 (3.05)	5 (4.59)	0.540
Chest CT scores (Initial diagnosis)				
Left lung	1 (0–2)	0 (0–2)	1 (1-3)	<0.001
Right lung	1 (0–2)	1 (0–2)	2 (1-4)	<0.001
Whole lung	2 (1-4)	2 (0–3)	4 (2-6)	<0.001

^*a*^ Data are presented as median (IQR) or *n *(%). *n* is the number of patients with available data. Percentages may not total 100 because of rounding. *P* < 0.05 were statistically significant.

## DISCUSSION

The frequent mutation of SARS-CoV-2 makes it more contagious and transmissible, which not only brings serious panic to human society but also hinders the development of the global economy (19, 20). The Omicron variant is a novel coronavirus variant (B.1.1.529) that emerged in Africa at the end of 2021 (WHO Update on Omicron. 28 November 2021.https://www.who.int/news/item/28-11-2021-update-on-omicron). It is the main strain circulating worldwide with stronger infectivity and immune escape (5, 21). In the last three years, to further control and prevent the spread and transmission of the Omicron variant, the global push for vaccination policies, and a total of 13.59 bn vaccine doses have been administered globally as of 26 November 2023 (https://covid19.who.int/). Regrettably, vaccination has also failed to eliminate the infection with the Omicron variant.

On 08 January 2022, the first non-imported Omicron variant infection case was reported in Tianjin, China (Tianjin Municipal Health Commission. https://wsjk.tj.gov.cn/ZTZL1/ZTZL750/YQFKZL9424/FKDT1207/202201/t20220109_5774785.html). August 2022, the SARS-CoV-2 Omicron BA.1, a dominating Omicron sublineage, was spreading wildly in Tianjin. As of 08 August 2022, a total of 33.183 million doses of SARS-CoV-2 vaccines and 13.337 million persons had been vaccinated in Tianjin (https://wsjk.tj.gov.cn/ZTZL1/ZTZL750/YQFKZL9424/wjwymjzqk/202209/t20220903_5978415.html). The proportion of residents in Tianjin who had been vaccinated to different degrees was about 93.14% (Tianjin Municipal Health Commission. https://wsjk.tj.gov.cn/ZTZL1/ZTZL750/YQFKZL9424/FKDT1207/202201/t20220102_5769364.html). The vaccination rate of the patients enrolled in this study was as high as 95.7%. Similar to the previous study (22), even if the full course of SARS-CoV-2 vaccination is completed, the Omicron BA.1 infection cannot be avoided.

Studies have shown that SARS-CoV-2 is susceptible to infecting individuals of all ages, while individuals over the age of 60, as well as comorbidities such as hypertension, diabetes, chronic respiratory diseases, and cardiovascular disease, are at higher infection risk (7, 23, 24). Hypertension is inflammatory diseases that might result in a dysregulated immune response and promoting hyperinflammation during SARS-CoV-2 infection. Several studies have reported the possible link between hypertension and COVID-19 (7–10). Previous studies stated that the interactions between ACE/ACE2 and miRNAs in hypertensive patients might increase the possibilities of SARS-CoV-2 infection (25, 26). Additionally, many hypertension patients use ACE2 inhibitors and angiotensin-receptor blockers (ARBs) that upregulate the ACE2 receptor (27, 28), which is also thought to be one of the reasons why older adults with hypertension may be at higher risk of SARS-CoV-2 infection and experience a more severe course. In view of the interaction between ACE2 and SARS-COV-2 and the effect of ACE2 in the pathogenesis of hypertension, there is some speculation that hypertension may be involved in the COVID-19 pathogenesis.

A 2018 study showed that a nationwide survey conducted from October 2012 to December 2015 in China showed that the overall prevalence of hypertension in adults (aged ≥18 years) was 23% (29). Furthermore, another previous multicenter study suggested that 30% of COVID-19 patients with a median age of 56 had previously coexisting hypertension (7). Hypertension prevalence increased with age and 63.1% among those aged 60 and over (30). However, there was a lack of clinical characteristics analysis of hypertension APs infected with Omicron BA.1 in China and revealing the impact of hypertension on APs with Omicron BA.1 infection was essential for the prevention and control and precise treatment of the COVID-19 disease. Therefore, this study focused on analyzing the clinical features of hypertension APs to provide some preventive and therapeutic references for hypertension APs in the wave of the Omicron BA.1 pandemic.

In this study, 512 Omicron BA.1-infected APs were enrolled and divided into hypertension and non-hypertension group. The differences in clinical characteristics and laboratory data were comparatively analyzed between the two groups. Our results indicate that the age and BMI levels of hypertension APs were significantly higher than that of non-hypertension APs. Previous findings suggested that elder age and high BMI were risk factors for the severity of COVID-19 (31, 32). Radzikowska et al. suggested that higher BMI and older age lead to higher expression of CD147‐related genes on immune cells, which potentially able to influence the development and the course of COVID‐19 (13). Obesity (BMI ≥30 kg/m^2^) is linked with reduced oxygen saturation of blood by compromised ventilation at the base of the lungs. Additionally, some other features of low-grade inflammation caused by obesity may also occur, such as abnormal secretion of cytokines, adipokines, and interferons leading to impaired immune response (33). Although another study showed that obesity was not a risk factor for COVID-19 in the early reports from China (34), a high number of COVID-19 cases was observed in the regions with more obese people from Europe and North America (35). Thus, it is needed to furtherly explore the relationship between obesity and COVID-19. A previous study reported that COVID-19 patients with older age, hypertension, and high LDH level were more likely to develop severe disease (36). In this study, we found that a higher proportion of hypertensive APs experienced cough, expectoration, and fatigue than non-hypertension APs. Additionally, hypertension APs had a significantly lower proportion of asymptomatic conditions while a visible higher proportion of mild and moderate conditions compared to non-hypertension APs. There was no obvious relationship between hypertension and severe conditions because few APs were diagnosed with severe disease. Even so, these results enough indicated that hypertension played adverse effects on the clinical symptoms and classifications of Omicron BA.1 infection.

Despite SARS-CoV-2 primarily affects the respiratory system, several studies have emphasized the effects of this viral infection on other organs (37–41). To further explore whether hypertension aggravates the effects of SARS-CoV-2 on organ functions, the liver, kidney, and myocardium function-associated indicators were analyzed. Surprisingly, hypertension had notable effects on the liver, kidney, and myocardium function-associated indicators of APs infected with Omicron BA.1, which indicated that hypertension would further aggravate the injuries of the liver, kidney, and myocardial function of Omicron BA.1-infected APs. Liver injuries and abnormal liver biochemistry were also reported in SARS, MERS, and now in COVID-19 infections. It implies that there is a relationship between abnormal liver enzyme secretion and coronavirus infection. The kidney has also been proposed as a potential target of SARS-CoV-2 (42). Acute kidney injury (AKI) observed in the COVID-19 cases (43), the incidence rate of AKI in hospitalized patients is over 20%, and the incidence of AKI patients admitted to intensive care is over 50% (44). Meanwhile, patients with renal diseases are more likely to suffer from COVID-19 infection due to an increase in ACE2 expression. Renal damage caused by the SARS-CoV-2 virus may be caused by the following mechanisms: virus direct damage to renal cells through ACE2, unbalanced RAS activation may produce harmful hemodynamic effects, pro-inflammatory cytokines and thrombotic states caused by viral infection (45). Additionally, myocardial injury is associated with adverse outcomes in COVID-19 patients (46, 47). Based on recent case reports of clinically suspected myocarditis in COVID-19 patients, myocarditis has been proposed as a potential mechanism of myocardial injury (48–50). Although myocardial injury is strongly associated with the severity of COVID-19, the underlying phenotype of myocardial injury in these patients remains abstruse. The pathogenesis of the liver, kidney, and myocardial function indicators abnormalities is unclear, and possible causes include direct SARS-CoV-2 infection, systemic inflammatory response, and drug toxicity (37, 38). This study supported that hypertension further aggravates the risk of the liver, kidney, and myocardial function injury of APs infected with Omicron BA.1. Therefore, more attention should be paid to the monitoring of the liver, kidney, and myocardial function parameters in Omicron BA.1-infected hypertensive APs to early recognize organ injury, which is crucial for mitigating the severity and mortality of COVID-19.

The immune system plays a vital role in maintaining immune homeostasis and inflammatory response throughout the body. The acute surge of multiple inflammatory cytokines after SARS-CoV-2 infection is one of the characteristics of COVID-19 patients, and the severity of new COVID-19 is positively correlated with inflammatory cytokine levels. In inflammation, there is an increase in the total number of white blood cells, especially neutrophils (36). Moreover, Chen et al. suggested that progressively elevated IL-6 levels are a risk factor for fatal outcomes with COVID-19, and CRP was also positively correlated with the severity and the clinical outcome of the disease (36, 51, 52). Similarly, our data indicated that hypertension APs not only had higher WBCs, neutrophils, basophils, IL-6, and CRP levels but also had lower virus-specific IgM levels than non-hypertension APs, which further immunologically confirms that hypertension had adverse effects on COVID-19 APs. Furthermore, Godoy et al. suggested that thromboembolism was another prominent clinical feature of COVID-19 patients and 5%–30% of in-hospital patients developed a clinically apparent thrombotic event (15). In this study, we found that hypertension APs had visible lower APTT and PT levels but higher PTA levels, which indicated that hypertension might cause APs to be in a procoagulant disease state. Previous research suggested that the hyper-coagulable disease state with a frequent occurrence of venous thromboembolic events (VTE) and pulmonary *in situ* thrombosis in COVID-19 patients was recently termed immune-thrombosis (18). COVID-19 patients may suffer from organ damage as well as from secondary bacterial or fungal superinfections due to the dysregulated immune and coagulation systems (9, 53, 54). Therefore, our study emphasized that inflammatory and coagulation**-**associated indicators should be particularly focused in Omicron BA.1-infected hypertensive APs to prevent COVID-19 deterioration.

Additionally, on account of its ability to rapidly evolve and mutate, the SARS-CoV-2 virus may never be eradicated, hence providing various directions of research due to human and virus cohabitation for a prolonged period. Although many researchers have conducted in-depth discussions on the SARS-CoV-2-related research in the past 3 years, it is important to note that the relationship between hypertension, a physiologically complex disease, and the susceptibility and severity of COVID-19 is complex and still being investigated.

Nonetheless, this study has a few limitations that need to be acknowledged. First, this was a retrospective and single-center study, and the sample size could be increased to represent a more generalized population. Furthermore, it would be better if our study included data on APs who are seriously ill due to COVID-19 infection. Finally, some uncontrolled confounding factors may also affect the results of the associated factors. Retrospective observational studies may lead to data bias due to various reasons such as data sources.

Despite the limitations, the data collected come from medical examinations and clinical analyses; therefore, the results of the study can be considered reliable and accurate. It is important to note that these limitations do not negate the importance of the findings, but rather highlight the need for more research to fully understand the relationship between hypertension and Omicron BA.1 infection.

## Data Availability

All data generated or analyzed during this study are included in this article. Further enquiries can be directed to the corresponding author, Hong Mu.
